# Development of a Three-Dimensional Finite Element Model of Thoracolumbar Kyphotic Deformity following Vertebral Column Decancellation

**DOI:** 10.1155/2019/5109285

**Published:** 2019-05-20

**Authors:** Tianhao Wang, Zhihua Cai, Yongfei Zhao, Guoquan Zheng, Wei Wang, Dengbin Qi, Diyu Song, Yan Wang

**Affiliations:** ^1^Southwest Hospital, Third Military Medical University, Chongqing 400038, China; ^2^Department of Orthopaedics, General Hospital of Chinese People's Liberation Army, Beijing 100853, China; ^3^School of Electromechanical Engineering, Hunan University of Science and Technology, Xiangtan 411201, China

## Abstract

**Background:**

Vertebral column decancellation (VCD) is a new spinal osteotomy technique to correct thoracolumbar kyphotic deformity (TLKD). Relevant biomechanical research is needed to evaluate the safety of the technique and the fixation system. We aimed to develop an accurate finite element (FE) model of the spine with TLKD following VCD and to provide a reliable model for further biomechanical analysis.

**Methods:**

A male TLKD patient who had been treated with VCD on L2 and instrumented from T10 to L4 was a volunteer for this study. The CT scanning images of the postoperative spine were used for model development. The FE model, simulating the spine from T1 to the sacrum, includes vertebrae, intervertebral discs, spinal ligaments, pedicle screws, and rods. The model consists of 509580 nodes and 445722 hexahedrons. The ranges of motion (ROM) under different loading conditions were calculated for validation. The stresses acting on rods, screws, and vertebrae were calculated.

**Results:**

The movement trend, peak stress, and ROM calculated by the current FE model are consistent with previous studies. The FE model in this study is able to simulate the mechanical response of the spine during different motions with different loading conditions. Under axial compression, the rod was the part bearing the peak stress. During flexion, the stress was concentrated on proximal pedicle screws. Under extension and lateral bending, an osteotomized L1 vertebra bore the greatest stress on the model. During tests, ligament disruption and unit deletion were not found, indicating an absence of fracture and fixation breakage.

**Discussion:**

A subject-specific FE model of the spine following VCD is developed and validated. It can provide a reliable and accurate digital platform for biomechanical analysis and surgical planning.

## 1. Introduction

The thoracolumbar kyphotic deformity (TLKD) is a kind of spinal deformity caused by various diseases, including trauma, ankylosing spondylitis, Pott's kyphosis, Scheuermann's disease, and degenerative scoliosis [1–5]. Severe low back pain, spinal cord injury, and sagittal imbalance due to TLKD could influence the quality of life. In such cases, spinal osteotomy surgery is often necessary to correct the deformity.

Several spinal osteotomy techniques have been described available for treating TLKD, including Smith-Petersen osteotomy (SPO), pedicle subtraction osteotomy (PSO), and vertebral column resection (VCR). Vertebral column decancellation (VCD) is a new technique, first described for the treatment of congenital kyphoscoliosis and Pott's kyphosis [6]. Since then, this technique has also been adopted in the treatment of rigid scoliosis and sharp angular spinal deformity [6, 7]. Previous studies have demonstrated that VCD is a reliable and effective option to manage TLKD [3, 7, 8], but biomechanical research that characterized the specific treatment effect is rarely reported.

Finite element (FE) analysis is a biomechanical research method that is preferred over cadaver experiments, due to limitations in the accuracy of measurements and of comparisons between construct loads and motions in the cadaver model [9–12]. An accurate FE model could help (i) simulate osteotomy and internal fixation accurately, (ii) perform biomechanical analysis repeatedly, and also (iii) plan operations and guide surgical procedures [13, 14].

The aim of this study was to develop an accurate FE model of the spine with TLKD following VCD and to provide a reliable model for further biomechanical analysis.

## 2. Material and Methods

### 2.1. Basic Information of the Volunteer

**Figure 1:**
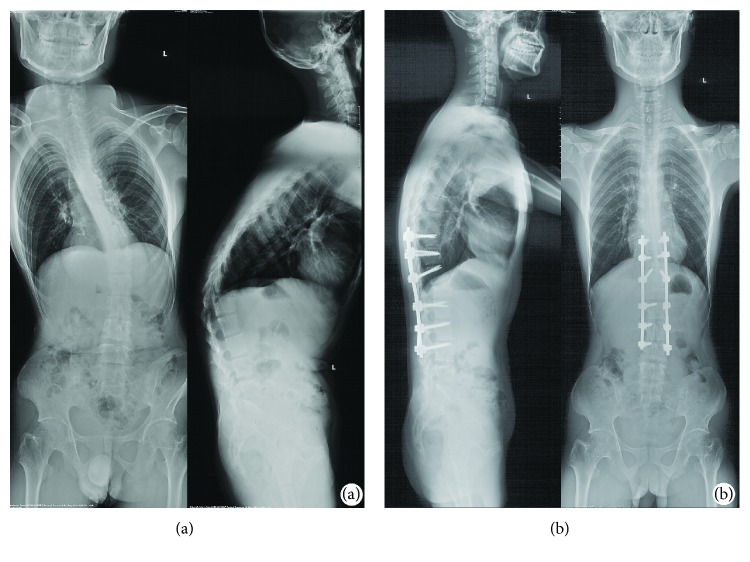
The full-length spine radiographs of a patient were obtained before surgery and one week after surgery. (a) Before surgery, the apical (kyphosis) vertebra was L1, with thoracolumbar kyphosis (T10 to L2) of 52°. The coronal Cobb angle between T9 and L3 was 56°. (b) Vertebral column decancellation was applied at L1, and the spine was fused from T10 to L4 with pedicle screws and a rod system. The thoracolumbar kyphosis and scoliosis were corrected to 11° and 12°, respectively.

CT scanning images of a male TLKD patient were used for developing an FE model. This patient volunteered to participate in this study (height 168 cm, weight 65 kg). The patient had a 12-year history of ankylosing spondylitis and kyphotic deformity for 5 years; there was no history of spinal fractures or other spine or joint surgeries. VCD was performed at the L1 vertebra. The segments from T10 to L4 were fused (Figure 1).

### 2.2. Construction of a Geometric Model

**Figure 2:**
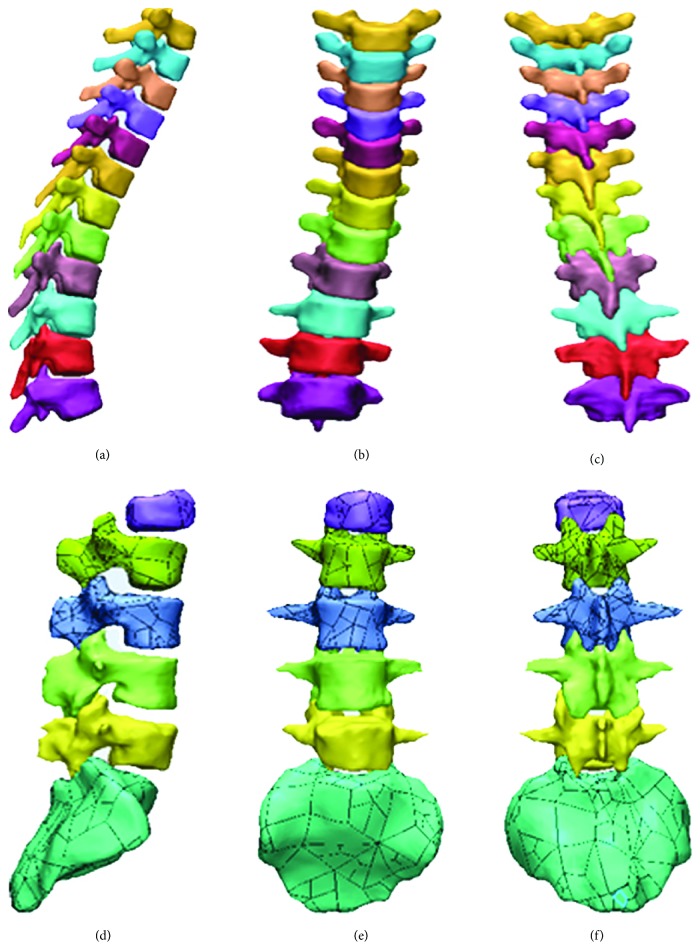
3D geometric model of the spine. (a), (b), and (c) are the thoracic spine, and (d), (e), and (f) are the lumbar spine and sacrum.

The DICOM data of CT images were obtained 1 week postoperatively. The slice thickness of CT images was 0.5 mm. A total number of 434 tomographic pictures were imported into MIMICS 17.0 (Materialise NV, Leuven, Belgium). These 2D images were converted to 3D point cloud data. Then, the 3D data were imported into 3-Matic 9.0 (Materialise NV, Leuven, Belgium) to generate a 3D geometric model of the spine (Figure 2).

### 2.3. Mesh Generation

The geometric model generated by the previous step was imported into ICEM-CFD (ANSYS Inc., Canonsburg, PA, USA). The blocks were created following the bottom-up method and grid projection method. This process was layer by layer like brick building: firstly, creating blocks; secondly, stretching faces; and then copying topology to create units. The structure was consistent with the structure of the vertebrae, screws, and rods.

### 2.4. Development of an Intervertebral Disc Model

**Figure 3:**
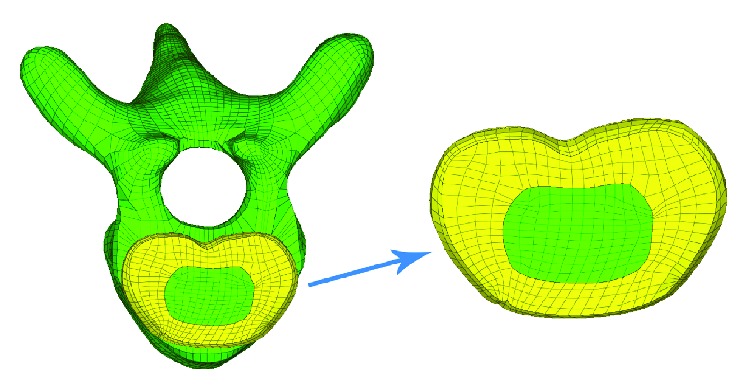
Finite element model of the intervertebral disc. The disc model consisted of four layers of fibrous rings and six layers of nucleus pulposus.

To simulate the structure and mechanotransduction, the model of intervertebral discs was optimized. The intervertebral disc model consisted of a four-layered annulus fibrosus and six-layered nucleus pulposus (about 37485 nodes and 27200 units). The units in the surfaces of intervertebral discs and adjacent endplates were individually associated (Figure 3).

### 2.5. Reservation of Pedicle Screw Paths

**Figure 4:**
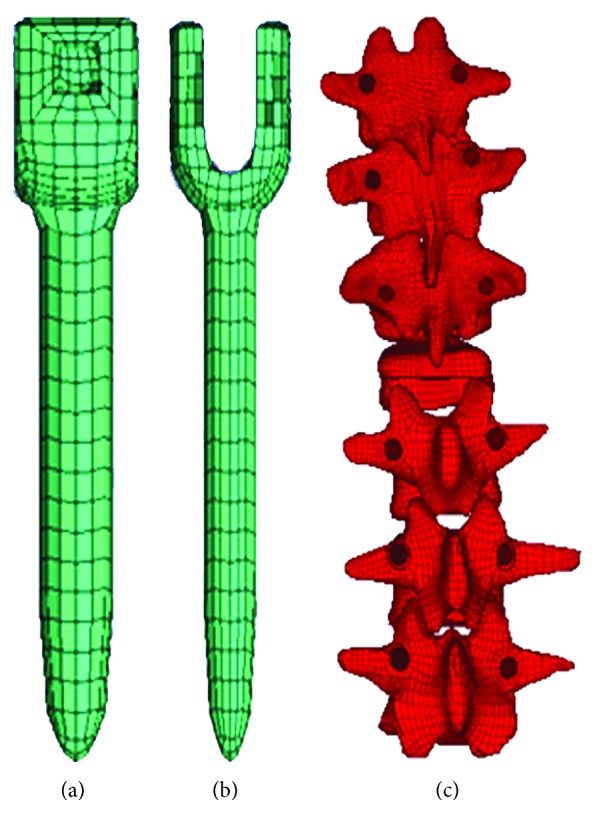
Meshed model of pedicle screws (a) and vertebrae with screw paths (b).

A pair of pedicle screws was inserted in each of the following vertebrae: T10, T11, T12, L2, L3, and L4. The geometric model of the pedicle screws was imported into Pro/Engineer (PTC Corporation, Needham, MA, US) to remove the thread. Then, the modified screw and vertebra models were imported into HyperMesh (Altair Engineering Inc., Troy, MI, USA) in an IGES format to remove the screw paths from the vertebrae by the Boolean operation. The hexahedron units around the screw paths were remeshed (Figure 4).

### 2.6. Establishment of an Entire Model

**Figure 5:**
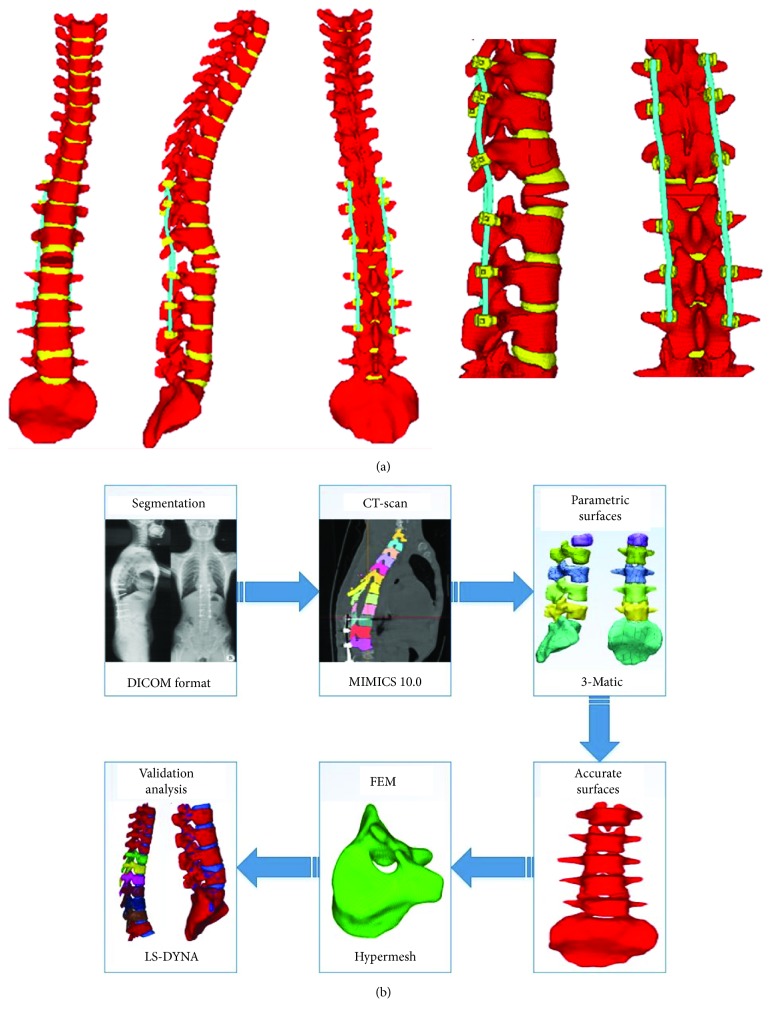
(a) The entire finite element model of the instrumented spine following vertebral column decancellation. (b) The flow chart of the development steps.

All the components, including thoracic vertebrae, lumbar vertebrae, intervertebral discs, pedicle screws, rods, interspinous ligaments, anterior longitudinal ligaments, posterior longitudinal ligaments, ligamentum flava, and supraspinal ligament, were included and meshed as a spring element. Finally, the entire model was established as depicted in Figure 5. The 3D FE model of the TLKD spine following VCD was well developed, including thoracic and lumbar vertebrae, intervertebral discs, paravertebral ligaments, pedicle screws, and rods. The FE model included 509580 nodes and 445722 hexahedral units (Table 1). Well, the whole procedure of the model meshing is shown in Figure 5(b).

### 2.7. Setting the Material Properties

The biomechanical parameters of different parts of the model were set based on former relevant literatures on human biomechanical models.  Table 2 demonstrates the material properties [15]. Tensile stress was observed to be subjected by ligaments, and the number of fibers was related to the magnitude of the force. In this paper, its mechanical properties were obtained by the ligament tensile test of Yoganandan et al. [16]. The elasticity coefficients of the anterior longitudinal ligaments, posterior longitudinal ligaments, interspinous ligaments, ligamentum flava, and the supraspinal ligament were 21.34 N/mm, 36.42 N/mm, 19.96 N/mm, 26.78 N/mm, and 10.04 N/mm, respectively.

Studies have shown that when the strain of the bone exceeds the yield strain, its elastic modulus decreases with the increase of the load. Therefore, the strength of the endplate is 1/3 of the cortical bone [15] and defined as ∗MAT_POWER_LAW_PLASTICITY. The water content of nucleus pulposus is as high as 70%-90% and defined as viscoelastic materials like ∗MAT_VISCOELASTIC; the annulus matrix is mainly composed of collagen fibers and simulated by an elastic material named ∗MAT_ELASTIC; the reinforced fiber membrane is simulated with the composite material ∗MAT_FABRIC referring to the uniaxial tensile test data of the fiber loop reinforcing fiber film of Holzapfel et al. [17].

The cartilage hardness and strength are closely related to the articular cartilage surface while facing tensile loading. Therefore, the cartilage and vertebrae are also connected with a common node to ensure accuracy of force transmission. And it is simulated by the elastic material ∗MAT_ELASTIC. The detailed material and unit properties of each organization are shown in Table 1.

The experiment showed that the stress-strain curve of the ligament is linear before the yield point. Therefore, the tensile load of the ligament is simulated by ∗MAT_SPRING_ELASTIC. The detailed data was obtained according to Wang et al. [18] as shown in Table 2. The_Surface_To_Surface Contact was used in the screws and vertebrae. And the screws and the rods are connected by a rigid body in hypermesh and were set in Automatic_Single_Surface.

### 2.8. FEM Validation

The intact model of segments from L2 to the sacrum was used for validation and was compared with reported experimental results [19, 20]. The freedom of the sacrum was constrained strictly. We applied a moment of 10 Nm to the upper lamina terminalis of the L2 vertebra to simulate different loads during flexion, extension, and lateral bending [18, 19].

### 2.9. Loading and Boundary Conditions

After validation, the FE model was proved to be reliable for further studies. The biomechanical responses of the osteotomized spine under different loading conditions were also analyzed. The freedom of the sacrum was constrained strictly. A fixed loading force of 300 N, simulating the gravity of upper body, was applied to the upper lamina terminalis of T1 to simulate axial compression [20]. A pure unconstrained bending torque of 10 Nm was applied to the upper lamina terminalis of the T1 endplate [18, 19]. Different motions of the spine including flexion, extension, and lateral bending were simulated.

## 3. Results

### 3.1. Results of Validation

Comparing the results obtained from the intact/uninstrumented model with data from the literature, we found that the ranges of motions (ROMs) as calculated by our model were consistent with the data from the literature. The calculated ROMs of each segment from L2 to L5 under different loading conditions are listed in Table 3.

### 3.2. Results of Simulation of Internal Fixation

**Figure 6:**
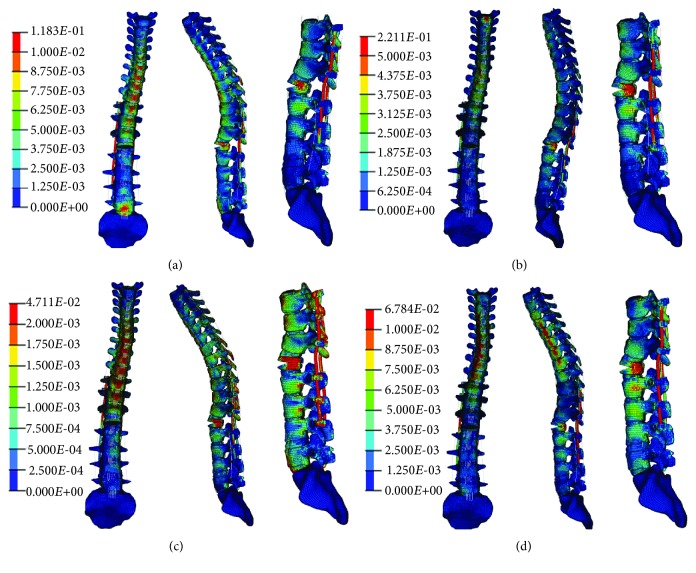
Stress nephograms of the model under different loading conditions: (a) axial compression, (b) flexion, (c) extension, and (d) lateral bending.

As shown in stress nephograms (Figure 6), under axial compression, the rod was the part bearing the peak stress. During flexion, the stress was concentrated on proximal pedicle screws. Under extension and lateral bending, an osteotomized L1 vertebra bore the greatest stress on the model. During tests, ligament disruption and unit deletion were not found, indicating an absence of fracture and fixation breakage.

## 4. Discussion

An osteotomized and instrumented TLKD spine FE model was developed and validated as a potential platform for the assessment of the initial and long-term effects of osteotomy and implants. There are several advantages of the FE model in the current study.

First, it is a long-segment model based on CT images from a postoperative patient. In the past spinal FE studies, the authors always developed an intact model from a healthy volunteer first and then added a fixation system to the model [21–23]. This method is convenient to develop a normal spine model, but when it comes to spinal deformity, the difficulty in simulating an abnormal spine curvature becomes a drawback. The current model is constructed from a postoperative patient with TLKD and therefore provides a better simulation condition that is realistic to clinical populations. Besides, in previous studies, the spinal segments included in the models were limited. Liu et al. [22] developed a lumbar spine model (L2 to sacrum) to investigate the biomechanical stability after different fusion procedures. Park et al. [24] developed a thoracolumbar spine model from T12 to S1 to study the effects of different fusion levels on the biomechanical properties of the spine. Bess et al. [9] developed a model from T7 to L5 to evaluate the protective effects of posterior polyester tethers for proximal junctional kyphosis. In these studies, the models only involved instrumented segments and lacked a simulation of the overall biomechanical response of the entire spine. The model in the current study involves vertebrae from T1 to the sacrum, and the stress and response in any part of the spine, under various force loadings, are clearly observed.

Second, the model was meshed into an all-hexahedron structure. In FE analysis, it is well accepted that a hexahedral mesh is better than a tetrahedral mesh. To analyze the same problem and obtain similar model accuracy, the number of tetrahedral units required is 10 times greater than that of hexahedral units. Hence, the computing time is considerably reduced. Furthermore, hexahedrons are more like the trabecular bone structure and provide better analysis accuracy. Also, because the shape of the vertebrae is irregular, tetrahedral units are more prone to suffer from aberration and remeshing, reducing analysis accuracy. However, hexahedral mesh generation is more complex than tetrahedral mesh generation, and none of the currently available automatic methods can generate an all-hexahedral mesh for any geometry so far. Nevertheless, hexahedral units are better than tetrahedral units. In previous studies, most of the models used a combination of tetrahedral units for vertebrae and hexahedral units for intervertebral discs [9, 22, 24]. The model developed in the current study, however, is meshed in hexahedron elements, which can give the model high simulation accuracy.

Third, an accurate model of the intervertebral discs and osteotomized vertebrae was established. The structure of the intervertebral disc is more complex than that of the vertebral body, comprising the annulus fibrosus and nucleus pulposus. Consistent with most of the previous studies, our intervertebral disc models have been developed with outer elements of the annulus fibrosus and inner elements of the nucleus pulposus and different material properties were defined. The intervertebral discs play an important role in force transmission of the spine. Therefore, the model of the intervertebral discs necessarily shows an ideal performance in mechanotransduction. In the current model, the elements on the surface of intervertebral discs match exactly with the elements on adjacent vertebral endplates, decreasing the loss of energy when a force is transmitted through the contact surface and rendering the analysis results more accurate. Besides, the model of the osteotomy site, the L2 vertebral body, is developed to simulate an idealized VCD model [3, 6]. This technique is a new, safe, and effective osteotomy strategy and provides satisfactory correction results. Biomechanical analysis for VCD is rarely conducted; the current FE model provides a reliable VCD simulation platform.

The current FE model was further verified by a comparison with previous studies. The intact lumbar model was used for the verification of the ROM. A spine FE model from T1 to sacrum was developed in our study. We chose the part of L2-L5 for validation due to the following reasons: Firstly, the lumbar spine has the largest range of motion and ensuring the effectiveness of lumbar segments is the most important. Secondly, the model was developed by the same method in each part. The positive validation result of L2 to L5 is able to indicate the effectiveness of the entire model. Thirdly, due to the difference of specimens and force loading condition, the biomechanical experiments on thoracic spine segments are lack of reference value for this study. Comparing with Shirazi-Adl et al. [20] and Yamamoto et al. [19] studies, the ROMs of L2–L3, L3–L4, and L4–L5 were similar in flexion, extension, and lateral bending. The slight differences between the studies are a result of the different loading and boundary conditions. However, for the three loading conditions, the movement trend, peak stress, and ROM calculated by the current FE model are consistent with previous studies. The FE model in this study is able to simulate the mechanical response of the spine during different motions with different loading conditions.

The effects of osteotomy and fixation are also studied. According to the stress nephogram, the locations of stress concentration under different loading conditions are clearly observed. During axial compression, the rods suffer peak stresses. When the patient is standing, the rods play a key role in keeping the spine vertical. Additionally, the stresses on the superior half of the rods are greater than those on the inferior half, because the thoracic spine is kyphotic and the center of gravity of the upper body is found ventral to the fixations. In most clinical reports, the pedicle screws are regarded as the weakest part of the posterior construct. Upon flexion, the stresses on the proximal pedicle screws are greater than those on the distal parts. Although the unit deletion, which indicates the occurrence of fracture and fixation failure, was not found in the screws and rod model during testing, it is still important to prevent (i) fixation breakage occurring in proximal areas and (ii) the occurrence of proximal junctional kyphosis. As for the vertebrae, the osteotomy site suffers maximum stress due to the smaller contact surface between the upper and lower half vertebrae, caused by osteotomy. The stress concentration at the osteotomy site could lead to compression fracture and cause a loss of correction. Therefore, necessary procedures to disperse stress on the construct are important to increase the safety of fixations and to persistently preserve the position correction of the spine.

There are several limitations in this study. A more realistic mode of force loading for the entire spine model is needed for further study. In this study, as in other studies, forces are loaded on the upper surface of the top vertebra. In short-segment spine models, this loading method is unrealistic. However, in long-segment models, the movement as simulated by the model is not identical to the actual movement of the spine; however, this loading method is sufficiently realistic to study the stiffness and the safety of an instrumented spine. A better method that can simulate the movement of the spine in a living human body is needed. Also, more biomechanical experiments are needed to measure different material properties of tissues in various diseases. The material properties set in this model were based on a healthy human body, but VCD can also be adopted in the case of disease, such as Pott's kyphosis and ankylosing spondylitis, during which the structural change of the spine and surrounding tissues occurs as the disease progresses. To study the surgery effect on patients with these diseases, more accurate material properties have to be set. The current model is based on a specific subject, but it is available to generalize the model to other patients and other types of diseases through changing the curvature of the spine or osteotomy vertebrae. The material properties are also can be adjusted to simulate different bone densities and different fixation materials.

## 5. Conclusions

An FE model of the spine following VCD is developed and validated. Our model can serve as a reliable and accurate digital platform for biomechanical analysis and surgical planning. After osteotomy and instrumentation, the maximum stress is inflicted on rods during axial compression, on proximal pedicle screws during flexion, and on the osteotomy site during extension and lateral bending. Further experiments must be conducted to find more appropriate force loading modes and more accurate material properties in different diseases.

However, the article did not fully verify the model due to the lack of experimental data on the thoracolumbar spine; at the same time, the follow-up work concentrates on the performance reducing the stress on the vertebrae of the transverse link and nail and only do relevant qualitative analysis. The requirements for the accuracy of the model are not harsh.

## Figures and Tables

**Table 1:** Number of nodes and hexahedrons of the model.

	Intervertebral discs	Rods	Screws	Cortical bone	Cancellous bone	Total
Nodes	37485	5002	11466	91652	363975	509580
Hexahedrons	27200	3840	6912	91646	316124	445722

**Table 2:** Material properties of the finite element model [18].

	Cortical bone	Cancellous bone	Endplate	Endplate cartilage	Nucleus pulposus	Annulus fibrosis	Cartilage
Element type	Shell	Hexahedron	Shell	Shell	Hexahedron	Hexahedron	Shell

Material model	Power-law plasticity	Power-law plasticity	Power-law plasticity	Isotropic elastic	Viscoelastic	Isotropic elastic	Isotropic elastic

Material properties	*ρ* = 1.83 g/cm^3^ *E* = 16700 MPa *v* = 0.3 *k* = 440.8 MPa *n* = 0.2772	*ρ* = 1.0 g/cm^3^ *E* = 291 MPa *v* = 0.3 *k* = 7.118 MPa *n* = 0.2741	*ρ* = 1.83 g/cm^3^ *E* = 5567 MPa *v* = 0.3 *k* = 146.9 MPa *n* = 0.2772	*ρ* = 1.68 g/cm^3^ *E* = 25 MPa *v* = 0.4	*K* = 2.2 GPa Gs = 2 MPa Gl = 1.4 MPa	*ρ* = 1.2 g/cm^3^ *E* = 3.4 MPa *v* = 0.49	*ρ* = 1.68 g/cm^3^ *E* = 10 MPa *v* = 0.4

*ρ* indicates the density. *E* indicates the Young modulus. *v* indicates the Poisson ratio. *k* indicates the strength coefficient. *n* indicates the strain hardening exponent. *K* indicates the volume model. Gs indicates the short-time shear modulus. Gl indicates the long-time (infinite) shear modulus.

**Table 3:** Comparison of the range of motion.

	Flexion (°)			Extension (°)			Lateral bending (°)		
	Model 1	Model 2	Model 3	Model 1	Model 2	Model 3	Model 1	Model 2	Model 3
L2–L3	3.86	5.4	3.28	2.97	3.3	2.32	4.77	5	3.31
L3–L4	3.06	6.1	3.58	2.12	2.3	1.18	3.47	4.3	3.33
L4–L5	3.58	7.1	4.49	2.56	4	3.89	4.01	3.8	2.08

Model 1 is the model developed in the current study. Model 2 is the model developed by Yamamoto et al. [19]. Model 3 is the model developed by Shirazi-Adl et al. [20].

## Data Availability

The data used to support the findings of this study are included within the article. If some researchers need more detailed data, it will be available from the corresponding authors upon request.
